# Long-term follow-up of a case of Coats disease in a 10-year-old boy with spontaneous peeling of preretinal macular fibrosis: a case report

**DOI:** 10.1186/s12886-022-02414-x

**Published:** 2022-04-27

**Authors:** Tomoka Mizobuchi, Takashi Nishiuchi, Yusaku Miura, Ken Fukuda

**Affiliations:** Department of Ophthalmology and Visual Science, Kochi Medical School, Kochi University, Kohasu, Oko-cho, Nankoku City, Kochi 783-8505 Japan

**Keywords:** Coats disease, Preretinal fibrosis, Posterior vitreous detachment, Optical coherence tomography

## Abstract

**Background:**

Coats disease is a retinal vascular disorder characterized by aneurysms and telangiectasias. Macular fibrosis is a complication of Coats disease that results in vision loss. Macular fibrosis rarely develops in the natural course and often occurs after treatment with intravitreal bevacizumab, photocoagulation, or cryotherapy. Here, we have described an unusual case of spontaneous peeling of preretinal macular fibrosis in a patient with untreated Coats disease.

**Case presentation:**

A 10-year-old Japanese boy presented with vision loss in his left eye. The patient’s left visual acuity was 20/28. Fundus examination of his left eye revealed thick preretinal macular fibrosis around the optic disc and macula. In addition, retinal telangiectasis, microaneurysms, hard exudates, and retinal hemorrhages were observed in the left peripheral temporal retina. We diagnosed his condition as Coats disease with preretinal macular fibrosis. Two months later, optical coherence tomography revealed preretinal macular fibrosis detachment at the foveal lesion without any treatment. During follow-up, preretinal macular fibrosis at the macular lesion was completely detached. Further, posterior vitreous detachment was observed and the shape of the macula and the patient’s left visual acuity had improved.

**Conclusions:**

In our case, both formation and spontaneous peeling of preretinal macular fibrosis occurred without any treatment for Coats disease, which is an unusual finding. Vitreous changes might have occurred during the natural clinical course, causing subsequent posterior vitreous detachment and resulting in spontaneous peeling of fibrosis.

## Background

Coats disease was first reported by George Coats in 1908 [1]. It is a retinal vascular disorder characterized by aneurysms and talengiectasias [2]. It often occurs unilaterally and is common in young men. Shields proposed the following stages of Coats disease: retinal telangiectasia only (Stage 1), telangiectasia with exudation (Stage 2), retinal detachment (Stage 3), and neovascular glaucoma (Stages 4 and 5) [2]. Treatment for Coats disease varies according to its stage—observation is recommended in mild cases, laser photocoagulation and cryotherapy are required in cases with stages 2–3 disease, and vitrectomy and glaucoma surgery are required in cases with advanced-stage disease.

Macular fibrosis is a complication of Coats disease that results in vision loss and includes preretinal fibrosis and epiretinal membrane [2]. Macular fibrosis is rarely observed in the natural course before treatment [3] and is often formed after treatment with intravitreal bevacizumab, photocoagulation, or cryotherapy [4–6].

Here, we have described an unusual case of spontaneous peeling of preretinal macular fibrosis in a patient with untreated Coats disease.

### Case presentation

**Fig. 1:**
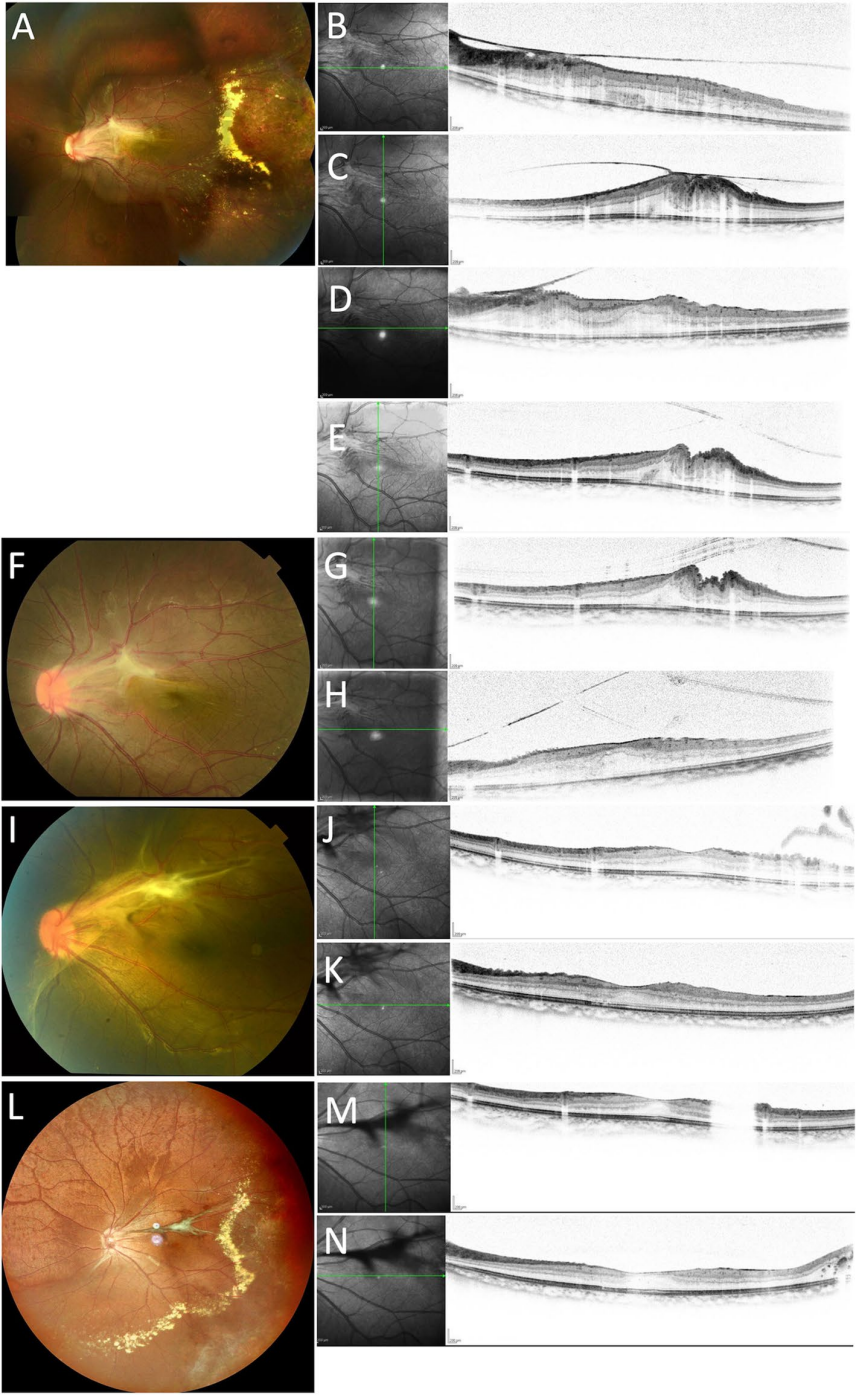
Fundus photographs and OCT images of the left eye. At initial presentation (A-C), fundus photograph (A) shows thick preretinal macular fibrosis from the optic disc to the macula. Retinal telangiectasis, microaneurysms, hard exudates, and retinal hemorrhage are observed. OCT image reveals severe distortion of the macula from preretinal fibrosis (B, C). At 2 months after the first visit (D, E), OCT image shows peeling off of the preretinal fibrosis at the foveal lesion. At 4 months after the first visit (F–H), fundus photograph (F) shows complete peeling off of preretinal fibrosis at the macular lesion but presence of retinal folds. OCT image shows retinal distortion (G, H). At 13 months after the first visit (I-K), fundus photograph (I) shows PVD expansion and peeled off preretinal macular fibrosis with PVD. OCT image shows an improved shape of the macula (J, K). At 4 years and 8 months after first visit, fundus photograph (L) shows complete peeling off of the fibrosis from the retina by PVD, which is visible as a floater attached to the posterior hyaloid membrane. OCT image (M, N) shows an almost-normal shape of the macula. OCT, optical coherence tomography; PVD, posterior vitreous detachment

A 10-year-old Japanese boy presented with vision loss in his left eye. The patient’s best-corrected visual acuity was 20/13 in the right eye and 20/28 in the left eye, and his intraocular pressure was within the normal limit. No abnormalities were observed in the anterior ocular segments. Fundus examination of the left eye revealed preretinal macular fibrosis around the optic disc and macula (Fig. 1A). Optical coherence tomography (OCT) revealed macular distortion due to preretinal macular fibrosis (Fig. 1B-C). In addition, retinal telangiectasis, microaneurysms, hard exudates, and retinal hemorrhages were observed in the left peripheral temporal retina. No abnormalities were observed in the right eye. Fluorescein fundus angiography could not be performed because the patient’s parents did not wish to have one. We diagnosed his condition as stage 2 Coats disease. We did not perform any treatment at initial presentation because no exudative or tractional retinal detachment was observed. Two months later, OCT revealed preretinal macular fibrosis detachment at the foveal lesion (Fig. 1D-E). Four months later, the preretinal macular fibrosis at the macular lesion was completely detached (Fig. 1F-H), and the patient’s left visual acuity had increased to 20/22. Posterior vitreous detachment (PVD) had expanded with complete peeling off of preretinal macular fibrosis and his macular formation improved (Fig. 1I-N). Although hard exudates expanded from the peripheral temporal to inferior regions, there was no exudative or tractional retinal detachment after 4 years and 8 months.

## Discussion and conclusions

Macular fibrosis in Coats disease includes preretinal fibrosis, vitreoretinal fibrosis, epiretinal membrane [6, 7], extramacular fibrosis [3], and subfoveal nodules [8], which can occur from the anterior retina to the sub-retina. Although macular fibrosis is observed in only 2% patients with Coats disease at initial presentation [9], it occurs in 23%–40% patients treated with intravitreal bevacizumab, photocoagulation, and cryotherapy during follow-up [3, 5, 7, 10]. Macular fibrosis due to intraretinal neovascularization leads to poor visual prognosis due to retinal layers involvement [8]. Preretinal or epiretinal fibrosis, is a risk factor for tractional retinal detachment and subsequent vision loss. Pars plana vitrectomy with membrane peeling is a treatment approach for macular fibrosis to prevent tractional retinal detachment and improve vision [6, 7].

Spontaneous detachment of macular fibrosis is also rare, but some cases of Coats disease with spontaneous peeling of macular fibrosis after photocoagulation have been reported [11, 12]. Vitreous changes might have occurred during the natural clinical course, causing subsequent PVD, which might have resulted in the spontaneous detachment of fibrosis. Macular fibrosis in these reported cases was probably due to the posterior vitreous membrane. Our patient also had premacular fibrosis consisting of a thick posterior vitreous membrane. Our patient had a very unusual course; both formation and spontaneous detachment of premacular fibrosis occurred without any treatment for Coats disease. The posterior vitreous membrane might have played an important role in both the formation and peeling of premacular fibrosis in Coats disease.

We have described a case of a young patient with vitreoretinal traction due to preretinal macular fibrosis resulting from untreated Coats disease. During the clinical course, preretinal macular fibrosis, thick posterior vitreous membrane detachment and spontaneous peeling off, and PVD were noted.

## Data Availability

Not applicable.

## Abbreviations

OCT: Optical coherence tomography
PVD: Posterior vitreous detachment
